# Associations between second-hand smoke exposure in pregnancy and age of childhood asthma development

**DOI:** 10.1186/1710-1492-8-S1-A4

**Published:** 2012-11-02

**Authors:** Elinor Simons, Teresa To, Rahim Moineddin, David Stieb, Sharon Dell

**Affiliations:** 1 The Hospital for Sick Children, Child Health Evaluative Sciences, Toronto, ON, Canada; 2 The Hospital for Sick Children, Division of Respiratory Medicine, Toronto, ON, Canada; 3 The University of Toronto, Department of Family and Community Medicine, Toronto, ON, Canada; 4 Health Canada, Ottawa, ON, Canada

## Background

Maternal smoking during pregnancy has been associated with an increased hazard of incident childhood asthma. We investigated the association between any second-hand smoke exposure in early life and childhood asthma development.

## Methods

In the Toronto Child Health Evaluation Questionnaire, parents of 5619 grades 1-2 students reported age of physician-diagnosed asthma development, exposure to maternal and household second-hand smoke during pregnancy and the first year of life, socio-demographic factors, and other early-life exposures such as mold and cockroach. Using Cox proportional hazard models, we evaluated the longitudinal associations between second-hand smoke exposure and age of asthma development.

## Results

Household second-hand smoke exposure prevalence was 8.3% during pregnancy and 10.6% in the first year of life; 15.5% of children developed asthma. After adjusting for sex, prematurity, being born in Canada and maternal asthma, children exposed to home second-hand smoke during pregnancy were more likely to develop asthma and developed asthma sooner [adjusted hazard ratio (HR) 1.36, 95% confidence interval (CI): 1.09, 1.70], even after excluding children whose mothers smoked in pregnancy (HR 1.53, 95% CI: 1.09, 2.14). The association strengthened (HR 1.88, 95% CI: 1.16, 3.02) after adjusting for home second-hand smoke exposure in the first year.

## Conclusions

Home second-hand smoke exposure during pregnancy is associated with an increased hazard of childhood asthma development, even if the mother is not a smoker. Recommendations for smoking cessation during pregnancy should focus on pregnant women and members of their households.

